# Breakfast quality and its sociodemographic and psychosocial correlates among Italian children, adolescents, and adults from the Italian Nutrition & HEalth Survey (INHES) study

**DOI:** 10.1186/s12937-024-00924-6

**Published:** 2024-02-19

**Authors:** Claudia Francisca Martinez, Emilia Ruggiero, Augusto Di Castelnuovo, Simona Esposito, Simona Costanzo, Chiara Cerletti, Maria Benedetta Donati, Giovanni de Gaetano, Licia Iacoviello, Marialaura Bonaccio, INHES Study Investigators

**Affiliations:** 1 Department of Epidemiology and Prevention, IRCCS NEUROMED, Via Dell’Elettronica, Pozzilli, IS 86077 Italy; 2 Mediterranea Cardiocentro, Naples, Italy; 3 Department of Medicine and Surgery, LUM University “Giuseppe Degennaro”, Casamassima, BA Italy

**Keywords:** Breakfast quality, Psychosocial factors, Sociodemographic factors

## Abstract

**Background:**

Breakfast quality, together with regularity of breakfast, has been suggested to be associated with cardiometabolic health advantages. We aimed to evaluate the quality of breakfast and its socioeconomic and psychosocial correlates in a large sample of the Italian population.

**Methods:**

Cross-sectional analyses on 7,673 adult and 505 children/adolescent regular breakfast eaters from the Italian Nutrition & Health Survey (INHES; 2010-2013). Dietary data were collected through a single 24-h dietary recall. Breakfast quality was assessed through the Breakfast Quality Index (BQI) combining intake of ten food groups, energy, and nutrients of public health concern, and potentially ranging from 0 to 10. The association of sociodemographic and psychosocial factors with BQI were analyzed by multivariable-adjusted linear regression models.

**Results:**

The average BQI was 4.65 (SD ± 1.13) and 4.97 (SD ± 1.00) in adults and children/adolescents, respectively. Amongst adults, older age (β = 0.19; 95%CI 0.06 to 0.31 for > 65 vs. 20–40 years) and having a high educational level (β = 0.13; 0.03 to 0.23; for postsecondary vs. up to elementary) were independent predictors of better breakfast quality, while men reported lower BQI (β = -0.08; -0.14 to -0.02 vs. women). Perceived stress levels at home and work and financial stress were inversely associated with BQI. Children/adolescents living in Central and Southern Italian regions had lower BQI compared to residents in Northern Italy (β = -0.55; -0.91 to -0.19 and β = -0.24; -0.47 to -0.01, respectively).

**Conclusions:**

In adults, breakfast quality was associated with age, sex, and educational level. Perceived stress levels were inversely associated with the quality of breakfast. In children/adolescents, a north-south gradient in breakfast quality was observed.

**Supplementary Information:**

The online version contains supplementary material available at 10.1186/s12937-024-00924-6.

## Introduction

Breakfast is widely considered to be a key component of a healthy diet. Regular breakfast consumption has been associated with weight control, visceral fat, better cognitive function, and a favourable cardio-metabolic health [1–5]. Besides breakfast regularity, breakfast composition is an important aspect of breakfast in relation with the daily dietary intake of nutrients [6–9]. Furthermore, there is an increasing interest in examining the association between breakfast quality and overall health [10–12]. However, the criteria for an ideal composition of breakfast including types and amounts of foods, nutrients, and energy is not well established. Several breakfast quality indexes have been mainly developed for children and adolescents, based on core food groups outlined in national dietary guidelines [13, 14] and very few propose to include nutrient criteria to define a high-quality breakfast [14, 15]. The Breakfast Quality Index (BQI) is a tool for assessing the nutritional quality of breakfast in epidemiological studies [16], based on the food groups and nutrients intake with the rationale of O’Neil, 2014 [15] and the scoring system proposed by Monteagudo, 2013 [14]. In adults, an overall good breakfast quality has been associated with a healthier cardiometabolic profile independent of overall diet quality [17, 18] and with the achievement of daily nutrient requirements [9, 16]. Observational studies of breakfast consumption in association with mental health are also emerging [10–12]. In addition to the regularity of breakfast intake, specific foods and the quality of breakfast could be crucial for achieving beneficial effects on mental health [10, 19, 20].

In Italy, studies on breakfast and health outcomes are scarce and they are mainly focused on children or adolescents [21, 22]. Among Italian adults, a higher typical breakfast food consumption was inversely associated with well-established cardiovascular disease risk factors [23]. However, in this study, breakfast composition was assessed on population-specific intakes rather than relying on evidence-based recommendations for nutrients or food groups for breakfast consumption. There is still a lack of comprehensive assessment of breakfast quality in the Italian population.

To fill this knowledge gap, we sought to assess breakfast quality in a large sample of Italian adults, children and adolescents, by taking advantage of the large population enrolled in the Italian Nutrition & HEalth Survey (INHES) Study from 2010 to 2013.

Additionally, we investigated sociodemographic and psychosocial factors associated with breakfast quality. This analysis is valuable for identifying specific population groups with suboptimal breakfast quality, and possibly for defining public health strategies to promote a high-quality breakfast at the population level.

## Materials and methods

### Study population and design

A cross-sectional study was conducted among participants of the INHES study, which is a 3-year telephone-based survey on nutrition and health specifically designed to collect information on dietary habits (*i.e.,* quality, quantity, food, and meal patterns), food choice determinants, and food health awareness of the Italian population according to geographical distribution, age, gender, and socioeconomic status. A total of 9,422 men and women aged ≥ 4 years throughout Italy were enrolled between November 2010 and November 2013. Details about this cohort have been previously described [24]. The sampling was distributed across four seasons (excluding Christmas, Easter, and mid-August periods), and the survey calendar was organized to capture an adequate proportion of weekdays and weekend days at the group level. The recruitment of participants was performed using computer-assisted-telephone-interviewing (CATI). Data on regularity of breakfast were obtained by the Food Propensity Questionnaire [25]; for the present analyses, we excluded participants with missing data on the regularity of breakfast (adults *n* = 98 (1.1%); children and adolescents *n* = 5 (0.9%), and those identified as breakfast skippers (adults *n* = 931 (10.5%); children and adolescents *n* = 63 (11.0%). After further exclusions, the analytical sample consisted of 7,673 adults (20-97 years) and 505 children/adolescents (5-19 years), identified as regular breakfast eaters and with complete dietary data. The flowchart for selection of the study participants is reported in Supplementary Fig. 1.

The INHES study was conducted according to the guidelines laid down in the Declaration of Helsinki, and all procedures involving human subjects were approved by the Ethical Committee of the Catholic University of Rome. Verbal informed consent was obtained from all subjects. Verbal consent was witnessed and formally recorded.

### Dietary assessment

Data on food intake was collected through a self-recorded diary, by using a computer-based single day 24-h dietary recall interview (24HR) software, and the Italian version of the European Food Propensity Questionnaire (EFPQ) [25, 26].

For every eating occasion in the 24HR, participants were asked to carefully record and recall (a) time and place of consumption; (b) a detailed description of foods (or beverages), and (c) quantity consumed and brand (for manufactured foods). Portion sizes were reported by individuals with the help of a picture booklet. If the participant was on a particular diet and if the consumption reported differed from their usual diet was registered. Individual food items and recipes reported by the participants were later matched with those available in the food list of the data management system INRAN-DIARIO 3.1 by a nutritionist during the interviews [26, 27]. The final output database included information for the daily consumption of the 2,000 single food items that were included in the software food list.

### Breakfast quality in adults

The Breakfast Quality Index (BQI) was computed for each participant according to the method proposed by Lopez-Pereira [16]. The index involves ten components including 4 food groups, energy, and 5 nutrients [14–16]. The score considered the intake but not the amounts consumed of cereals and derivatives, fruits or vegetables, or dairy products. If the participant did not report consumption of the food group, the individual scored zero. No points were removed for unhealthy foods consumed at breakfast. One component scored positively if any combination of cereals, dairy products, and fruit or vegetables was consumed at breakfast (Supplementary Table 1).

Energy intake compliance between 15–25% of total daily energy intake (1 component) and nutrient intake (5 components) were based on quantitative criteria [15]. For nutrients, 1 point was assigned when the following criteria were met: (a) free sugar intake at breakfast < 10% total daily energy divided by the number of participants’ daily eating occasions (EO); (b) calcium intake ≥ 20% of the recommended dietary allowance according to participants’ life stage group as indicated by Italian Dietary Reference Intakes [28]; (c) saturated fat content < 10% total daily energy divided by the number of daily EO; (d) fibre intake > 25 g divided by the number of participants’ EO; and (e) sodium intake < 2000 mg divided by the number of daily EO. For analysis purposes, the BQI was further categorized as low (BQI between 0 and 3), medium (4-6), and high (7-10), as done in prior studies [16, 19].

### Breakfast quality in children/adolescents

For children and adolescents, the BQI was estimated according to the work of Monteagudo and colleagues [14], scoring one point each for consumption of cereals and derivatives, dairy products, fruit/vegetables, and monounsaturated fats (MUFA) (olive oil, vegetable oil); one point for intake of added sugar < 5% of total daily energy (sugar, jam, honey), MUFA: saturated fat ratio above the median for the population, energy intake providing 20–25% of total daily energy intake, and calcium intake between 200-300 mg at breakfast; one point for the absence of butter and margarine; and one point if cereals, fruit, and dairy products were included in the same meal. Scores on the BQI ranged from 1 to 10, Supplementary Table 2. For analyses purposes, the BQI was ranked into population-specific thirds, reflecting low (BQI from 0 to 4), medium (5), and high breakfast quality (6-10).

### Assessment of sociodemographic factors and covariates

Educational level was based on the highest qualification attained and was categorized as up to elementary school (corresponding to ≤ 5 years of study), lower secondary (> 5 ≤ 8 years), upper secondary (> 8 ≤ 13 years), post-secondary (> 13 years). Present occupation was grouped into non-manual worker, manual worker, housewife, retired, student and unemployed. Marital status was defined as married/living in a couple, single, separated/divorced, and widowed. Urban or rural environments were defined based on the urbanization level as described by the European Institute of Statistics (EUROSTAT definition) and obtained by using the tool ‘Atlante Statistico dei Comuni’ provided by the Italian National Institute of Statistics. Geographical areas included Northern (42%), Central (17.2%), and Southern (40.8%) Italian regions. Participants were classified as never (who has never smoked, or who has smoked less than 100 cigarettes in the lifetime), current (smoking one or more cigarettes per day at the time of interview), former (who had quit smoking at the time of interview) or occasional smokers (smoking less than 1 cigarette per day at the time of interview). Sport activity was self-reported (no/yes). History of cardiovascular disease and cancer, and previous diagnosis of diabetes, hypercholesterolemia, and hypertension were self-reported and categorized as no/yes. Body mass index (BMI) was calculated by using self-reported measurements of height and weight, calculated as kg/m^2^, and grouped into three categories normal (≥ 18.5 ≤ 24.9 kg/m^2^), overweight (≥ 25 ≤ 29.9 kg/m^2^), or obese (≥ 30 kg/m^2^). BMI in children/adolescents was categorised according to specific values for children considering sex and age [29].

### Ascertainment of psychosocial factors in adults

Information on psychosocial conditions during the previous 12 months was obtained by administering a standard set of questions to the adult sample of the INHES Study [30].

Self-rated health was assessed through a one-item question (“In general, how would you rate your health status”) and responses were arranged along a four-item Likert-type scale from ‘excellent’ to ‘poor’. Major adverse life events (yes/no) were assessed by asking participants whether, in the past year, they had experienced one or more of the following: (1) marital separation or divorce; (2) business failure; (3) major intra-family conflict; (4) death or major illness of a close family member; (5) loss of job or retirement, violence; (6) death of a spouse; (7) major personal injury or illness or (8) other major stress. Psychological distress was assessed through two items relating to stress at work and home, by asking participants how often in the past year they had felt stressed by indicating one of the following response options: (1) never; (2) sometimes; (3) most of the times; (4) often; (5) always.

Financial stress was self-reported in three levels (1) little or none; (2) moderate; or (3) high.

### Statistical analysis

Characteristics of study participants are described across thirds of the BQI in both adults and kids. Values are presented as numbers and percentages for categorical values and mean with standard deviation (SD) for continuous variables**.** Beta-coefficients with 95% confidence intervals (95%CIs) from multivariable-adjusted linear regression analyses were used to evaluate the association of sociodemographic and psychosocial factors with the BQI (continuous).

We fitted two multivariable- models: (1) Model 1 was adjusted for age, sex, and total daily energy intake; (2) Model 2 as in Model 1 and further controlled for geographical area, place of residence, educational level, occupation, marital status, smoking status, sport activity, BMI and previous history of cardiovascular disease, cancer, hypertension, hypercholesterolemia or diabetes.

Multinomial adjusted logistic regression models were used to derive odds ratios (ORs) and corresponding 95% CIs for participants in the medium or high BQI category both compared to the low BQI category.

Missing data on socioeconomic and psychosocial factors and covariates was lower than 3% in adults and less than 1% in the group of children/adolescents (Supplementary Fig. 1), and were handled using multiple imputation (SAS PROC MI, followed by PROC MIANALYZE) to maximise data availability for all variables, avoid bias introduced by not-at-random missing (MNAR) data patterns and achieve robust results over different simulations (*n* = 10 imputed datasets). Statistical hypotheses were tested using a two-tailed *P* < 0.05 level of significance. Data analysis was generated using SAS/STAT software, version 9.4 (SAS Institute Inc., Cary, NC, USA).

## Results

The analyses were conducted on 7,673 adults (54.6% women), with an average (SD) age of 57.1 (14.8) years, and 505 children and adolescents (47.3% girls) having a mean age of 14.4 (3.7) years. We identified a total of 25 foods and beverages consumed at breakfast that were then categorized into eight main food/beverage groups (Supplementary Table 3).

### Breakfast composition and quality among adults (20–97 years)

**Fig. 1:**
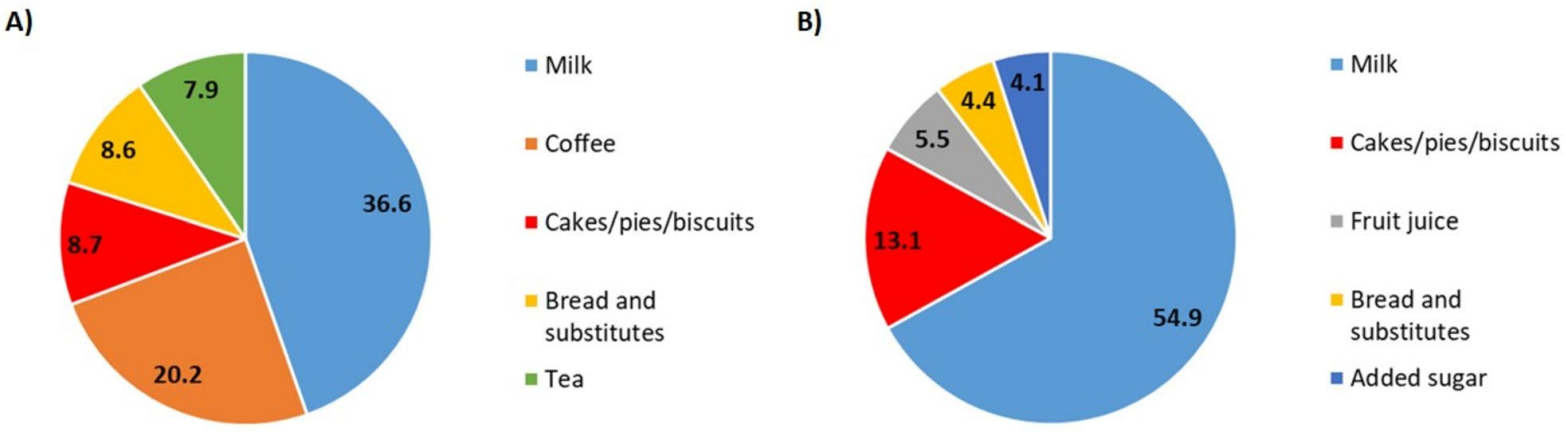
Top five contributing foods and beverages (g/d) to breakfast composition (g/d) in (**A**) adults (*n* = 7,673), and (**B**) children/adolescents (*n* = 505) from the INHES Study, Italy 2010-2013. INHES, Italian Nutrition & HEalth Survey

In adults, the top five foods and beverages (g/d) contributing to the total amount of food consumed at breakfast (g/d) were milk (36.6%), coffee (20.2%), cakes/pies/biscuits (8.7%), bread and substitutes (8.6%), and tea (7.9%) (Fig. 1A). From the total population 72% reported having coffee at breakfast, 61.7% consumed milk, 44.1% bread and substitutes, and 38.7% consumed cakes/pies and biscuits. The intake of added sugar was reported by 56.7% of participants. Only a small percentage of breakfast eaters reported consuming fruits (6.7%), fruit juices (3.8%), yogurt (8.1%), or jam and honey (8.7%) (Supplementary Table 4).

Table 1 shows the proportion of Italian adults scoring 1 point for each BQI component in the total population and across increasing categories of the BQI. Cereal and derivatives group was the most prevalent food component scored positively (79.1%), followed by dairy products (68.7%), and fruit or vegetables (6.9%). Only 2.7% of adults obtained one point for inclusion of a combination of three food groups (cereal, fruit or vegetables, and dairy products) together at breakfast. Most of the population did not comply with the nutrient criteria for fibre or calcium intake, whereas a large proportion of adults (80% or higher) met the criteria for sugar, saturated fat and sodium content.

**Table 1:** Individual components of the Breakfast Quality Index (BQI) and distribution of adult participants from the INHES study scoring 1 point for each component across BQI categories

		**Categories of BQI**			
	Total population	Low (0–3 points)	Medium (4–6 points)	High (≥ 7 points)	*p*-value
N of participants (%)	7673 (100)	1061 (13.8)	6190 (80.7)	422 (5.5)	
BQI (mean ± SD)	4.65 ± 1.13	2.91 ± 0.28	4.76 ± 0.72	7.34 ± 0.67	< 0.0001
Cereals and derivatives consumption	79.1	32.5	85.7	98.8	< 0.0001
Fruit or Vegetables consumption	6.9	1.7	5.4	42.2	< 0.0001
Dairy products consumption	68.7	33.0	72.8	99.3	
Cereal, Fruit or Vegetables and Dairy products consumption in the same meal	2.7	0.0	0.6	40.3	< 0.0001
Compliance with energy intake recommendations (15-25% of total daily energy)	20.8	2.1	20.1	78.2	< 0.0001
Free sugar content (< 10% total daily energy divided by the number of daily eating occasion of the individual)	82.1	40.1	88.4	94.3	< 0.0001
Calcium content (≥ 20% of daily value)	18.1	0.9	16.6	82.5	< 0.0001
Saturated fat content (< 10% total daily energy divided by the number of daily eating occasion of the individual)	88.7	85.7	89.0	91.7	0.001
Total fibre content (> 25 g divided by the number of daily eating occasion of the individual)	1.7	0.3	1.2	12.8	< 0.0001
Sodium content (< 2000 mg divided by the number of daily eating occasions of the individual)	96.2	95.2	96.5	94.8	0.034

INHES, Italian Nutrition & HEalth Survey

Values are percentages, unless otherwise indicated

*P*-values for differences across BQI categories were assessed by a generalized multivariable linear regression model

Cereals and derivatives include bread and substitutes; breakfast cereals; other cereals; cakes/pies/biscuits

Fruit or vegetables consumption include any type of fruit, nuts and vegetables

Dairy products include milk; yoghurt; cheese; other milk products and cocoa drinks

The average BQI (SD) in this Italian population of adults was 4.65 (1.13) (range 2 to 10) points. Only 5.5% of the participants were classified as having a high breakfast quality (BQI ≥ 7 points), 80.7% of the population had an average adherence (BQI 4-5 points), and 13.8% of participants fell into the bottom category (BQI 0-3 points).

All BQI individual components were disproportionally distributed across BQI categories. More than 90% of the population with high BQI levels included cereals or derivatives, and dairy products, but less than 50% met the fruits or vegetables criterion. The BQI component of optimal calcium intake was satisfactorily achieved only by participants scoring ≥ 7 points; a small proportion (12.8%) of participants in the high BQI category satisfied the fibre criterion (Table 1). Compared to participants in the highest BQI category, those in the bottom BQI group had lower energy intake both at breakfast and overall, and consumed more tea, coffee, and fruit juice; however, they were more likely to consume lower amounts of all foods, except for meat, fish, and eggs, snacks, vegetarian/vegan foods, and nut spread (Supplementary Table 4).

### Breakfast composition and quality among children and adolescents (5-19 years)

The top five contributing foods and beverages (g/d) to the total food consumed at breakfast (g/d) by children/adolescents were milk (54.9%), cakes/pies/biscuits (13.1%), fruit juice (5.5%), bread and substitutes (4.4%), and added sugar (4.1%) (Fig. 1B).

A large proportion of young participants consumed milk (75.0%), coffee (18.8%), cocoa/energy drinks (16.8%), and fruit juices (7.9%), while food groups mostly consumed were cakes/pies/biscuits (49.3%), bread and substitutes (18.8%), and breakfast cereals (9.7%). Only 4.5% of the sample reported fruit consumption at breakfast, while 30.1% of young participants reported consumption of added sugar (Supplementary Table 5).

The mean (SD) BQI of Italian children and adolescents was 4.97 (± 1.00); more than 90% of this sample scored 1 point for consumption of sugary products < 5% of total daily energy and for the absence of SFA and trans-rich fats (Table 2). A good proportion of study participants positively scored for cereals and derivatives (68.3%), and dairy products (78.6%) intake at breakfast, while consumption of fruits or vegetables was low (11.5%) (Table 2). Participants in the high BQI category had higher breakfast energy intake compared to those in the bottom category but did not report higher total energy intake (Supplementary Table 5). Differences across BQI categories were mostly found for intakes of tea, soft drinks, cocoa/energy drinks, nuts, jam/honey, and butter/margarine that were higher among young people scoring low on BQI compared to the high BQI group. High breakfast quality was associated with greater consumption of bread and substitutes, cakes/pies/biscuits, vegetable fat, and nut spread (Supplementary Table 5).

**Table 2:** Individual components of the Breakfast Quality Index (BQI) and distribution of children/adolescent from the INHES Study scoring 1 point for each component across BQI categories

		**Categories of BQI**			
	Total Population	Low (0–4 points)	Medium (5 points)	High (≥ 6 points)	*p*-value
N of participants (%)	505 (100%)	166 (32.9%)	191 (37.8%)	148 (29.3%)	
BQI (mean ± SD)	4.97 ± 1.00	3.84 ± 0.42	5.0 ± 0.0	6.19 ± 0.54	< 0.0001
Cereals and derivatives consumption	68.3	17.5	93.7	92.6	< 0.0001
Fruit or Vegetables consumption	11.5	9.6	5.8	20.9	< 0.0001
Dairy products consumption	78.6	79.5	72.8	85.1	0.021
Foods rich in simple sugars (sugar, jam, honey) < 5% of total daily energy	97.4	94.0	99.0	99.3	0.0028
MUFA-rich fats (olive oil, vegetable oil)	2.8	0.0	1.6	7.4	0.0001
MUFA:SFA ratio above the median	49.7	8.4	50.8	94.6	< 0.0001
Compliance with energy intake recommendations (20–25% of total daily energy)	9.3	1.8	2.6	26.3	< 0.0001
Cereals, fruit and dairy product in the same meal	2.0	0.0	0.0	6.8	< 0.0001
Calcium intake (200-300 mg)	79.2	79.5	73.8	85.8	0.026
Absence of SFA and trans-rich fats (butter, margarine)	97.8	93.4	100.0	100.0	< 0.0001

INHES, Italian Nutrition & HEalth Survey

Values are percentages, unless otherwise indicated

*P*-values for differences across BQI categories were assessed by a generalized multivariable linear regression model

Cereals and derivatives include bread and substitutes; breakfast cereals; other cereals; cakes/pies/biscuits

Fruit or vegetables consumption include any type of fruit, nuts and vegetables

Dairy products include milk; yoghurt; cheese; other milk products and cocoa drinks

*MUFA* monounsaturated fats, *SFA* saturated fats

### Sociodemographic and psychosocial correlates of breakfast quality

In linear regression models adjusted only for age, sex and total daily energy intake (Table 2; Models 1), sociodemographic factors associated with higher BQI were older age (β = 0.25; 95%CI 0.17 to 0.34 for participants aged > 65 years vs. 20-40-year group), urban residence (β = 0.11; 0.04 to 0.18 vs. rural areas) and being retired (β = 0.09; 0.003 to 0.17 vs. non-manual workers). Higher BQI levels were also observed among former smokers (β = 0.11; 0.04 to 0.17 vs. non-smokers) and for participants reporting some sport activities (β = 0.18; 0.12 to 0.25 vs. no sport activity), and for those with diabetes (β = 0.16; 0.06 to 0.25). BQI was found lower in men (β = -0.07; -0.12 to -0.02), and among individuals living in Southern Italy (β = -0.06; -0.12 to -0.01) (Table 1; Models 1).

In multivariable-adjusted regression models including all sociodemographic factors simultaneously, older age (β = 0.19; 95%CI 0.06 to 0.31 for participants aged > 65 years vs. 20–40-year group) remained associated with higher BQI, and being men was still linked to lower BQI levels (β = -0.08; 95%CI -0.14 to -0.02 vs. women).

Manual workers (β = 0.09; 0.01 to 0.17), housewives (β = 0.16; 0.06 to 0.26), retired (β = 0.12; 0.03 to 0.21), and unemployed participants (β = 0.18; 0.02 to 0.34) reported higher BQI levels compared to non-manual workers, as well as participants with postgraduate education (β = 0.13; 0.03 to 0.23 vs. up to elementary group) (Table 3; Model 2).

**Table 3:** Sociodemographic factors associated with Breakfast Quality Index (BQI) in adult participants from the INHES study, Italy 2010-2013

			**Categories of BQI**^**1**^						
	Total population (%)	BQI (mean ± SD^*^	Low (0–3 points) 1,061 (13.8)	Medium (4–6 points) 6,190 (80.7)	High (≥ 7 points) 422 (5.5)	β (95%CI)^1^	*p*-value^1^	β (95%CI)^2^	*p*-value^2^
Age groups (years)									
20–40	888 (11.6)	4.52 ± 1.10	146 (13.8)	710 (11.5)	32 (7.6)	Ref	Ref	Ref	Ref
41–65	4187 (54.6)	4.62 ± 1.12	600 (56.5)	3380 (54.6)	207 (49.0)	0.10 (0.02 to 0.18)	0.012	0.08 (-0.01 to 0.18)	0.080
> 65	2598 (33.8)	4.74 ± 1.15	315 (29.7)	2100 (33.9)	183 (43.4)	0.25 (0.17 to 0.34)	< .0001	0.19 (0.06 to 0.31)	0.0031
Sex									
Women	4187 (54.6)	4.64 ± 1.15	620 (58.4)	3331 (53.8)	236 (55.9)	Ref	Ref	Ref	Ref
Men	3486 (45.4)	4.65 ± 1.10	441 (41.6)	2859 (46.2)	186 (44.1)	-0.07 (-0.12 to -0.02)	0.0095	-0.08 (-0.14 to -0.02)	0.0052
Geographical area									
Northern Italy	3220 (42.0)	4.67 ± 1.17	438 (41.3)	2583 (41.7)	199 (47.2)	Ref	Ref	Ref	Ref
Central Italy	1318 (17.2)	4.72 ± 1.13	170 (16.0)	1064 (17.2)	84 (19.9)	0.04 (-0.03 to 0.11)	0.27	0.04 (-0.03 to 0.12)	0.22
Southern Italy	3135 (40.8)	4.60 ± 1.08	453 (42.7)	2543 (41.1)	139 (32.9)	-0.06 (-0.12 to -0.01)	0.023	-0.05 (-0.11 to 0.002)	0.056
Place of residence									
Rural	1042 (13.6)	4.55 ± 1.13	175 (16.5)	816 (13.2)	51 (12.1)	Ref	Ref	Ref	Ref
Urban	6631 (86.4)	4.66 ± 1.13	886 (83.5)	5374 (86.8)	371 (87.9)	0.11 (0.04 to 0.18)	0.0029	0.07 (-0.01 to 0.14)	0.070
Educational level									
Up to elementary	1393 (18.2)	4.71 ± 1.08	168 (15.9)	1152 (18.6)	73 (17.3)	Ref	Ref	Ref	Ref
Lower secondary	1960 (25.5)	4.60 ± 1.10	270 (25.5)	1595 (25.8)	95 (22.5)	-0.05 (-0.13 to 0.03)	0.24	-0.05 (-0.13 to 0.03)	0.25
Upper secondary	3055 (39.8)	4.63 ± 1.14	448 (42.1)	2446 (39.5)	161 (38.2)	0.02 (-0.06 to 0.10)	0.66	0.03 (-0.05 to 0.12)	0.43
Postsecondary	1265 (16.5)	4.71 ± 1.21	175 (16.5)	997 (16.1)	93 (22.0)	0.09 (-0.002 to 0.19)	0.055	0.13 (0.03 to 0.23)	0.014
Occupation									
Non-manual	2397 (31.2)	4.57 ± 1.15	404 (38.1)	1865 (30.1)	128 (30.3)	Ref	Ref	Ref	Ref
Manual	1289 (16.8)	4.61 ± 1.06	161 (15.2)	1078 (17.4)	50 (11.8)	0.02 (-0.05 to 0.10)	0.54	0.09 (0.01 to 0.17)	0.031
Housewife	831 (10.8)	4.68 ± 1.07	103 (9.7)	694 (11.2)	34 (8.1)	0.08 (-0.01 to 0.18)	0.078	0.16 (0.06 to 0.26)	0.0022
Retired	2819 (36.8)	4.73 ± 1.16	350 (33.0)	2272 (36.7)	197 (46.7)	0.09 (0.003 to 0.17)	0.042	0.12 (0.03 to 0.21)	0.0076
Student	121 (1.6)	4.57 ± 1.10	22 (2.0)	95 (1.6)	4 (1.0)	0.02 (-0.20 to 0.23)	0.85	0.10 (-0.12 to 0.32)	0.40
Unemployed	216 (2.8)	4.66 ± 1.05	21 (2.0)	186 (3.0)	9 (2.1)	0.12 (-0.03 to 0.28)	0.12	0.18 (0.02 to 0.34)	0.025
Marital status									
Married /in couple	5774 (75.2)	4.66 ± 1.13	778 (73.2)	4682 (75.6)	314 (74.4)	Ref	Ref	Ref	Ref
Unmarried	1105 (14.4)	4.57 ± 1.15	182 (17.2)	872 (14.1)	51 (12.1)	-0.01 (-0.10 to 0.07)	0.75	-0.02 (-0.11 to 0.06)	0.63
Separated/divorced	236 (3.1)	4.75 ± 1.09	21 (2.1)	198 (3.2)	17 (4.0)	0.12 (-0.02 to 0.27)	0.098	0.10 (-0.05 to 0.24)	0.19
Widowed	558 (7.3)	4.67 ± 1.14	80 (7.5)	438 (7.1)	40 (9.5)	-0.03 (-0.13 to 0.07)	0.58	-0.02 (-0.13 to 0.08)	0.66
Smoking habit									
Non-smoker	4778 (62.3)	4.63 ± 1.14	725 (68.3)	3799 (61.4)	254 (60.2)	Ref	Ref	Ref	Ref
Current	1052 (13.8)	4.56 ± 1.06	145 (13.7)	862 (13.9)	45 (10.7)	-0.04 (-0.12 to 0.03)	0.24	-0.04 (-0.11 to 0.04)	0.31
Former	1694 (22.0)	4.76 ± 1.13	172 (16.2)	1407 (22.7)	115 (27.2)	0.11 (0.04 to 0.17)	0.0013	0.09 (0.03 to 0.16)	0.0051
Occasional	149 (1.9)	4.74 ± 1.23	19 (1.8)	122 (2.0)	8 (1.9)	0.11 (-0.07 to 0.30)	0.22	0.12 (-0.06 to 0.30)	0.20
Sport activity									
No	6216 (81.0)	4.62 ± 1.11	894 (84.2)	4994 (80.7)	328 (77.7)	Ref	Ref	Ref	Ref
Yes	1457 (19.0)	4.77 ± 1.20	167 (15.8)	1196 (19.3)	94 (2.3)	0.18 (0.12 to 0.25)	< .0001	0.18 (0.11 to 0.24)	< .0001
Cardiovascular disease									
No	741 (96.6)	4.65 ± 1.13	1041 (98.1)	5966 (96.4)	409 (96.9)	Ref	Ref	Ref	Ref
Yes	257 (3.4)	4.73 ± 1.03	20 (1.9)	224 (3.6)	13 (3.1)	0.04 (-0.10 to 0.18)	0.19	0.02 (-0.12 to 0.16)	0.79
Cancer									
No	7420 (96.7)	4.64 ± 1.13	1034 (97.5)	5978 (96.6)	408 (96.7)	Ref	Ref	Ref	Ref
Yes	253 (3.3)	4.73 ± 1.15	27 (2.5)	212 (3.4)	14 (3.3)	0.07 (-0.07 to 0.21)	0.21	0.05 (-0.09 to 0.19)	0.45
Hypertension									
No	5186 (67.6)	4.62 ± 1.14	760 (71.5)	4153 (67.1)	273 (64.7)	Ref	Ref	Ref	Ref
Yes	2487 (32.4)	4.70 ± 1.11	301 (28.5)	2037 (32.9)	149 (35.3)	0.01 (-0.04 to 0.07)	0.62	-0.002 (-0.06 to 0.06)	0.96
Hyperlipidaemia									
No	6000 (78.2)	4.63 ± 1.13	864 (81.5)	4807 (77.7)	329 (78.0)	Ref	Ref	Ref	Ref
Yes	1673 (21.8)	4.70 ± 1.11	197 (18.5)	1383 (22.3)	93 (22)	0.03 (-0.03 to 0.09)	0.30	0.01 (-0.05 to 0.07)	0.75
Diabetes									
No	7078 (92.2)	4.63 ± 1.14	1016 (95.8)	5663 (91.5)	399 (94.5)	Ref	Ref	Ref	Ref
Yes	595 (7.8)	4.81 ± 0.98	45 (4.2)	527 (8.5)	23 (5.5)	0.16 (0.06 to 0.25)	0.0014	0.16 (0.06 to 0.26)	0.0015
Body mass index									
Normal weight	3755 (48.9)	4.61 ± 1.18	577 (54.4)	2960 (47.8)	218 (51.7)	Ref	Ref	Ref	Ref
Overweight	2916 (38.0)	4.67 ± 1.10	382 (36.0)	2377 (38.4)	157 (37.2)	0.02 (-0.03 to 0.08)	0.37	0.03 (-0.02 to 0.09)	0.23
Obese	1002 (13.1)	4.72 ± 1.03	102 (9.6)	853 (13.8)	47 (11.1)	0.06 (-0.02 to 0.14)	0.12	0.07 (-0.01 to 0.16)	0.079

INHES, Italian Nutrition & HEalth Survey

Values presented are n and proportions unless otherwise indicated

^1^Beta coefficient, 95% confidence intervals (95% CI) and *p*-values from a linear regression model including age groups, sex and total daily energy intake (kcal/d)

^2^Beta coefficient, 95% confidence intervals (95% CI) and *p*-values from a linear regression model including all the variables listed in the table simultaneously, and further controlled for total energy intake (kcal/d)

^*^Raw

In multivariable-fully adjusted models (Table 4; Model 2), participants with increasing levels of psychological stress at home (*i.e.,* often/always) tended to have lower BQI (β = -0.28; 95%CI -0.50 to -0.09) compared to those who reported no stress at all (Table 4; Model 2). The same inverse trend was observed for perceived stress at work (β = -0.24; 95%CI -0.46 to -0.01 for ‘often’ vs. ‘never’). High levels of financial stress were also linked to lower BQI compared to participants reporting little or no stress related to financial issues (β = -0.27; -0.45 to -0.08) (Table 4; Model 2). Decreasing levels of self-rated health was associated with higher BQI levels, whereas adverse life events were not related to breakfast quality (Table 4; Models 2).

**Table 4:** Psychosocial factors associated with the Breakfast Quality Index (BQI) in adult participants from the INHES Study, Italy 2010-2013

		**Breakfast Quality Index (BQI)**				
*Psychosocial factors*	N of participants (%)	BQI (mean ± SD)*	β (95%CI)^1^	*p*-value^1^	β (95%CI)^2^	*p*-value^2^
**Self-rated health status**						
Excellent	1211 (15.8)	4.30 ± 1.18	Ref	Ref	Ref	Ref
Good	4962 (64.7)	4.71 ± 1.11	0.38 (0.31 to 0.46)	< .0001	0.38 (0.31 to 0.45)	< .0001
Fair	1405 (18.3)	4.74 ± 1.12	0.41 (0.32 to 0.51)	< .0001	0.42 (0.32 to 0.51)	< .0001
Poor	95 (1.2)	4.64 ± 1.06	0.32 (0.08 to 0.55)	< .0001	0.32 (0.08 to 0.56)	< .0001
**Adverse life events**						
None	6836 (89.1)	4.65 ± 1.13	Ref	Ref	Ref	Ref
At least one	837 (11.9)	4.67 ± 1.13	0.04 (-0.04 to 0.12)	0.29	0.03 (-0.05 to 0.11)	0.52
**Stress at home**						
Never	205 (2.8)	4.83 ± 1.09	Ref	Ref	Ref	Ref
Sometimes	4320 (55.4)	4.62 ± 1.16	-0.19 (-0.35 to -0.03)	0.017	-0.17 (-0.33 to -0.01)	0.032
Most of the times	2871 (38.2)	4.69 ± 1.09	-0.15 (-0.30 to 0.01)	0.071	-0.14 (-0.30 to 0.02)	0.085
Often/always	277 (3.6)	4.53 ± 1.00	-0.30 (-0.50 to -0.10)	0.0040	-0.28 (-0.50 to -0.09)	0.0047
**Stress at work**^**a**^						
Never	149 (1.9)	4.90 ± 1.07	Ref	Ref	Ref	Ref
Sometimes	1503 (19.6)	4.54 ± 1.18	-0.28 (-0.47 to -0.09)	0.0038	-0.22 (-0.42 to -0.03)	0.022
Most of the times	2077 (27.1)	4.62 ± 1.08	-0.23 (-0.42 to -0.05)	0.014	-0.17 (-0.36 to 0.02)	0.082
Often	326 (4.2)	4.52 ± 1.07	-0.30 (-0.52 to -0.08)	0.0066	-0.24 (-0.46 to -0.01)	0.038
Always	142 (1.9)	4.69 ± 1.22	-0.13 (-0.39 to 0.13)	0.31	-0.10 (-0.36 to 0.17)	0.47
Not working	3476 (45.3)	4.71 ± 1.13	-	-	-	-
**Financial stress**^**b**^						
Little or none	151 (2.0)	4.87 ± 1.11	Ref	Ref	Ref	Ref
Moderate	4382 (57.1)	4.71 ± 1.13	-0.15 (-0.33 to 0.03)	0.10	-0.14 (-0.32 to 0.04)	0.14
High	2898 (37.8)	4.55 ± 1.14	-0.30 (-0.49 to -0.12)	0.0012	-0.27 (-0.45 to -0.08)	0.0041
Non-responders	242 (3.1)	4.54 ± 1.01	-	-	-	**-**

INHES, Italian Nutrition & HEalth Survey

Values presented are n and proportions unless otherwise indicated

^a^Analysis run on 4,197 after exclusion of non-worker participants

^b^Analysis run on 7,431 after exclusion of non-responders

^1^Beta coefficient, 95% confidence intervals (95% CI) and *p*-values from a linear regression model including age groups, sex and total daily energy intake (kcal/d)

^2^Beta coefficient, 95% confidence intervals (95% CI) and *p*-values from a linear regression model including age groups, sec, total daily energy intake (kcal/d), geographical area, place of residence, educational level, occupation, marital status, smoking status, sport activity, cardiovascular disease, cancer, hypertension, hypercholesterolemia, diabetes, and body mass index

^*^Raw means

For children/adolescents, a geographical gradient in BQI was observed, with young participants living in Central (β = -0.55; 95%CI -0.91 to -0.19) and Southern (β = -0.24; 95%CI -0.47 to -0.01) Italian regions reporting poorer breakfast quality compared to their counterparts residing in Northern Italy (Table 5). No further sociodemographic differences were recorded.

**Table 5:** Sociodemographic factors associated with the Breakfast Quality Index (BQI) in children/adolescents from the INHES study, Italy 2010–2013

			**Categories of BQI**						
	N of subjects (n total = 505)	BQI (mean ± SD)	Low (0–4 points) *n* = 166; 32.9%	Medium (5 points) *n* = 191; 37.8%	High (≥ 6 points) *n* = 148; 29.3%	β (95%CI)^1^	*p*-value^1^	β (95%CI)^2^	*p*-value^2^
Age groups (years)									
5-12	135 (26.7)	4.94 ± 0.92	45 (27.1)	51 (26.7)	39 (26.3)	Ref	Ref	Ref	Ref
13 19	370 (73.3)	4.96 ± 1.03	121 (72.9)	140 (73.3)	109 (73.7)	0.03 (-0.18 to 0.23)	0.79	0.22 (-0.12 to 0.56)	0.20
Sex									
Girls	239 (47.3)	4.94 ± 0.99	77 (46.3)	90 (47.1)	72 (48.6)	Ref	Ref	Ref	Ref
Boys	266 (52.7)	4.99 ± 1.01	89 (53.6)	101 (52.9)	76 (51.4)	0.05 (-0.12 to 0.23)	0.55	0.03 (-0.15 to 0.21)	0.75
Geographical area									
Northern Italy	107 (21.2)	5.21 ± 1.06	24 (14.5)	41 (21.5)	42 (28.4)	Ref	Ref	Ref	Ref
Central Italy	44 (8.7)	4.64 ± 1.04	21 (12.6)	15 (7.8)	8 (5.4)	-0.56 (-0.92 to -0.21)	0.0018	-0.55 (-0.91 to -0.19)	0.0028
Southern Italy	354 (70.1)	4.93 ± 0.96	121 (72.9)	135 (70.7)	98 (66.2)	-0.29 (-0.51 to -0.07)	0.0092	-0.24 (-0.47 to -0.01)	0.039
Place of residence									
Rural	61 (12.1)	4.80 ± 0.81	24 (14.5)	25 (13.1)	12 (8.1)	Ref	Ref	Ref	Ref
Urban	444 (87.9)	4.99 ± 1.02	42 (85.5)	166 (86.9)	136 (91.9)	0.18 (-0.09 to 0.46)	0.19	0.10 (-0.18 to 0.39)	0.47
Educational level									
Up to elementary	180 (35.6)	4.99 ± 0.91	54 (32.5)	73 (38.2)	53 (35.8)	Ref	Ref	Ref	Ref
Lower/upper secondary	325 (64.4)	4.95 ± 1.05	112 (67.5)	118 (61.8)	95 (64.2)	-0.22 (-0.54 to 0.10)	0.096	-0.23 (-0.55 to 0.09)	0.16
Smoking habit									
Non-smoker	448 (88.7)	4.98 ± 0.96	142 (85.5)	173 (90.6)	133 (89.9)	Ref	Ref	Ref	Ref
Current/former/occasional	57 (11.3)	4.88 ± 1.25	24 (14.5)	18 (9.4)	15 (10.1)	-0.12 (-0.41 to 0.16)	0.40	-0.05 (-0.34 to 0.23)	0.70
Sport activity									
No	170 (33.7)	4.89 ± 0.91	62 (37.3)	61 (31.9)	47 (31.8)	Ref	Ref	Ref	Ref
Yes	335 (66.3)	5.00 ± 1.04	104 (62.7)	130 (68.1)	101 (68.2)	0.10 (-0.08 to 0.29)	0.27	0.08 (-0.11 to 0.27)	0.42
Body mass index									
Normal weight	419 (83.0)	4.97 ± 1.02	137 (82.5)	156 (81.7)	126 (85.1)	Ref	Ref	Ref	Ref
Overweight/Obese	86 (17.0)	4.94 ± 0.91	29 (17.5)	35 (18.3)	22 (14.9)	-0.04 (-0.28 to 0.19)	0.71	0.01 (-0.23 to 0.25)	0.96

INHES, Italian Nutrition & HEalth Survey

Values presented are n and proportions unless otherwise indicated

^1^Beta coefficient, 95% confidence intervals (95% CI) and *p*-values from a linear regression model including age groups, sex and total daily energy intake (kcal/d)

^2^Beta coefficient, 95% confidence intervals (95% CI) and *p*-values from a linear regression model including all the variables listed in the table simultaneously, and further controlled for total energy intake (kcal/d)

^*^Raw means

Similar findings were obtained from multinomial logistic regression models, both in adults and children/adolescents, although most associations were significant only when the extreme categories of the BQI were compared (Supplementary Table 6-8). Logistic regression analyses in adults confirmed that main predictors of a high breakfast quality were older age (OR = 2.42; 95%CI 1.32–4.43 for age > 65 years vs. 20–40 years) and higher educational attainment (OR = 2.02; 95%CI 1.30 -3.15 for postgraduate vs. lowest educational level), and reinforced the strength of the association between the BQI with geographical area (OR of being the in the high BQI category = 0.72; 95%CI 0.55-0.95 for Southern vs. Northern Italy). Men had lower likelihood of having a high breakfast quality (OR = 0.76; 95%CI 0.58-0.99 vs. women). Living in urban areas was linked to increased likelihood of having a medium BQI (OR = 1.23; 95%CI 1.02-1.49). Being separated/divorced, former smokers and reporting physical activity were associated with higher odds of being in the medium and high BQI categories (Supplementary Table 6). Chronic health conditions (*i.e.,* history of CVD, diabetes and obesity) were only linked to higher likelihood of reporting a medium BQI (Supplementary Table 6). The directions of the associations of BQI with psychosocial factors remained unchanged, although the strengths were attenuated in some cases (Supplementary Table 7).

For children/adolescents, data from multinomial logistic regression analyses confirmed that participants living in central and southern Italian regions were less likely to have a high quality breakfast compared to those from the Northern areas (OR = 0.22; 95%CI 0.08-0.59 and OR = 0.54; 95%CI 0.29-0.98, respectively), and residing in central Italy was also inversely associated with reporting a medium BQI (OR = 0.39; 95%CI 0.16-0.93), as well as having a higher educational level (OR = 0.41; 95%CI 0.17-0.96 for lower/secondary vs. up to elementary) (Supplementary Table 8).

## Discussion

In Italian adults, higher breakfast quality (BQI ≥ 7 points) was reported by 5.5% of participants, whereas 13.8% of the population was classified as having poor breakfast quality (BQI 0-3). Data from European or North America Countries lacking, these findings are in line with data from Brazil and Iran: in the National Dietary Survey in Brazil including 22,279 adults, only 6% of participants were found to have an optimal breakfast quality [16]; whereas, in a small Iranian sample about 10% of the population was identified as highly compliant to a high breakfast quality [31].

The top food and beverages contributing to breakfast composition in our sample of Italian adults were milk, coffee, cakes /pies/biscuits and bread and substitutes, confirming prior data from a large Italian population [23].

Individual food items of the BQI mostly prevalent in Italian adults were cereals and derivatives and dairy products, while only a small proportion (6.9%) of participants usually consumed fruit or vegetable at breakfast; major differences with data from a Brazilian population [16] were observed for positive scoring on saturated fat and sodium content, with more favourable estimates among Italians.

Amongst children/adolescents, the average BQI of 4.97 was lower than values found in a representative sample of Brazilian school children aged 8-17 years, reporting a mean BQI of 5.64 [14], but higher compared to a sample of Spanish children and adolescents with an average BQI of 4.29 [32]. Also, analyses in this Brazilian cohort revealed differences in the quality of breakfast by sex and age, while in our study we only observed a geographical gradient in BQI.

Differences in the quality of breakfast could be determined by several factors, including sociodemographic and psychosocial factors, as already observed in prior studies highlighting a sociodemographic gradient in diet quality worldwide [33–36]. We found that older age, being woman and having a high educational level were independent predictors of higher breakfast quality in adults. Occupation was also linked to the quality of breakfast, although the direction of the association was somehow counterintuitive, since non-manual and unqualified workers tended to report a higher BQI than non-manual workers.

We observed that participants with higher breakfast quality were also more likely to report other health-impacting behaviours, such as sport activity and no-smoking status; this is in line with prior epidemiological evidence indicating that health-related behaviours typically tend to cluster [37]. Also, we found that participants with diabetes had a better quality of breakfast, and this is possibly due to *e.g.,* nutritional advice for glucose control (including having breakfast every day, not skipping meals, and healthy eating) that is given to people living with type 2 diabetes [38].

Our analyses on sociodemographic determinants are consistent with previous data on German-speaking Swiss residents, suggesting that higher education level, being a woman and reporting to be fit were related to a healthier breakfast composition [39]; a socioeconomic gradient in breakfast quality was also found in the Brazilian population [16], whereas a study on Iranian participants [31], in contrast with our data, did not observe relevant sociodemographic differences in BQI, with the exception of age, with younger participants having higher breakfast quality than older subjects. The key role of education in diet quality is well-established [40] and potential explanations include a good set of knowledge and skills to make healthier food choices, that possibly determine a higher breakfast quality.

Another important finding was the inverse association between breakfast quality with financial stress, and perceived stress at home and at work among adults. Previous studies on breakfast consumption have suggested an inverse relationship between several mental health outcomes such as stress and anxiety [41, 42], cognitive failure [43] and depression [44, 45]. However, breakfast quality has been scarcely studied in relation to mental health outcomes. Findings from an observational study among adolescents from Spain suggested that participants eating a good quality breakfast had higher scores for several dimensions of health-related quality of life, and lower stress and depression compared to participants having poor quality breakfasts [10]. Similarly, a cross-sectional survey including 3,480 adolescents from Greece reported a favourable association of mental health with breakfast quality [46].

While there is not a clear biological mechanism linking breakfast consumption directly to mental health, studies have indicated that a higher intake of fruits and vegetables within the overall diet is associated with reduced odds of experiencing worries, tension, and a lack of joy in adults, independently of other lifestyle factors [20]. Several nutrient-dense foods such as whole grains, eggs, and dairy products are rich in nutrients involved in mental health, endogenous serotonin production, and mood regulation such as magnesium, calcium, tryptophan [47, 48], and choline [49].

Further longitudinal studies are warranted to assess the directionality of the association between breakfast quality and mental well-being, and possibly define what type of food included at breakfast could favourably impact mental health.

### Strengths and limitations

This study is possibly the first to evaluate the quality of the Italian breakfast in adults and children/adolescents, and to examine its major sociodemographic and psychosocial correlates.

Major strengths include the large population sample consisting of more than 8,000 adults, children and adolescents recruited throughout Italy. Furthermore, we used a novel methodology to evaluate the quality of breakfast in population studies adopted for purposes of comparison between populations.

Several limitations should also be addressed. First, causality or directionality are limited by the cross-sectional design; we cannot exclude the possibility of a reverse causality bias that could potentially explain, *e.g.,* the inverse association between self-rated health with breakfast quality. Also, residual confounding by unmeasured factors cannot be fully ruled out.

Secondly, self-reported dietary data are susceptible to bias and error, including social desirability and recall bias, imprecision in assessing portion sizes and inadequacies in food composition. Moreover, height and weight were self-reported, and this is prone to criticisms, including over or under-reporting. However, data were collected by trained interviewers, and beforehand a short photograph atlas and guidance notes to estimate food portion sizes were delivered to participants. Thirdly, the use of a single 24 HR is another weakness since it might not completely reflect the usual dietary intakes and potentially leads to biased estimates; nevertheless, a single 24HR could be sufficient to identify average consumption in a target population [50–52].

Another limitation is that dietary data were collected almost a decade ago, thus might not reflect the current dietary intakes in the Italian population, although being the most updated data available so far in the Country, and in line with timeframes from the majority of studies in the field [6, 31, 32, 53, 54]; moreover, the analyses on correlates of BQI are independent of the time of data collection.

Finally, the generalizability of the findings is limited to the Italian population; however, these results contribute to the scarce body of knowledge on breakfast quality, and its sociodemographic and psychosocial correlates.

## Conclusions

In conclusion, data from this large nutrition survey indicate that only a small proportion of Italian adults, children, and adolescents have a high breakfast quality. Major deficiencies were observed for fruit and vegetable consumption, compliance with energy recommendations, and fibre content at breakfast. In adults, breakfast quality varied across age groups, sex, and educational level, and was also linked to stress-related indicators, suggesting that effective public health policies should specifically address the nutritional needs of more vulnerable population groups. Future studies with up-to-date dietary data are warranted to understand the diverse breakfast-related nutritional challenges of the Italian population and to possibly confirm the association with sociodemographic and psychosocial correlates to implementing interventions and strategies to improve breakfast quality, preferably since childhood.

### Supplementary Information

**Additional file 1:** **Supplementary Figure 1. **Flowchart for selection of study participants from the INHES Study, Italy 2010-2013. **Supplementary Table 1.** Criteria for scoring the Breakfast Quality Index in adult participants from the INHES Study, Italy 2010-2013. **Supplementary Table 2.** Criteria for scoring the Breakfast Quality Index in children and adolescents from the INHES Study, Italy 2010-2013. **Supplementary Table 3.** Food groups and corresponding food items for scoring the breakfast quality indices in adults and children/adolescents from the INHES Study, Italy 2010-2013. **Supplementary Table 4. **Foods and beverages consumption at breakfast across BQI categories, in adult participants (20-97 years) from the INHES Study, Italy 2010-2013. **Supplementary Table 5. **Foods and beverages consumption at breakfast across BQI categories, in children/adolescent (5-19 years) from the INHES Study, Italy 2010-2013. **Supplementary Table 6. **Sociodemographic factors associated with Breakfast Quality Index (BQI) categories in adult participants from the INHES Study, Italy 2010-2013, by means of adjusted odds ratios (OR) with 95%CI. **Supplementary Table 7. **Psychosocial factors associated with the Breakfast Quality Index (BQI) in adult participants from the INHES Study, Italy 2010-2013, by means of adjusted odds ratios (OR) with 95%CI. **Supplementary Table 8. **Sociodemographic factors associated with the Breakfast Quality Index (BQI) categories in children/adolescents from the INHES Study, Italy 2010-2013, by means of adjusted odds ratios (OR) with 95%CI. Supplementary appendix.

## Data Availability

The data underlying this article will be shared on reasonable request to the corresponding author. The data are stored in an institutional repository (https://repository.neuromed.it) and access is restricted by the ethical approvals and the legislation of the European Union.
