# Supplementary material for: Breakfast quality and its sociodemographic and psychosocial correlates among Italian children, adolescents, and adults from the Italian Nutrition & HEalth Survey (INHES) study
Source: Nutr J. 2024 Feb 19;23:20. doi: 10.1186/s12937-024-00924-6 (PMC10875855; doi:10.1186/s12937-024-00924-6)
Supplement: Supplementary file 1 — Additional file 1: Supplementary Figure 1. Flowchart for selection of study participants from the INHES Study, Italy 2010-2013. Supplementary Table 1. Criteria for scoring the Breakfast Quality Index in adult participants from the INHES Study, Italy 2010-2013. Supplementary Table 2. Criteria for scoring the Breakfast Quality Index in children and adolescents from the INHES Study, Italy 2010-2013. Supplementary Table 3. Food groups and corresponding food items for scoring the breakfast quality indices in adults and children/adolescents from the INHES Study, Italy 2010-2013. Supplementary Table 4. Foods and beverages consumption at breakfast across BQI categories, in adult participants (20-97 years) from the INHES Study, Italy 2010-2013. Supplementary Table 5. Foods and beverages consumption at breakfast across BQI categories, in children/adolescent (5-19 years) from the INHES Study, Italy 2010-2013. Supplementary Table 6. Sociodemographic factors associated with Breakfast Quality Index (BQI) categories in adult participants from the INHES Study, Italy 2010-2013, by means of adjusted odds ratios (OR) with 95%CI. Supplementary Table 7. Psychosocial factors associated with the Breakfast Quality Index (BQI) in adult participants from the INHES Study, Italy 2010-2013, by means of adjusted odds ratios (OR) with 95%CI. Supplementary Table 8. Sociodemographic factors associated with the Breakfast Quality Index (BQI) categories in children/adolescents from the INHES Study, Italy 2010-2013, by means of adjusted odds ratios (OR) with 95%CI. Supplementary appendix. [file 12937_2024_924_MOESM1_ESM.docx]

**Supplementary Figure 1.** Flowchart for selection of study participants from the INHES Study, Italy 2010-2013

**
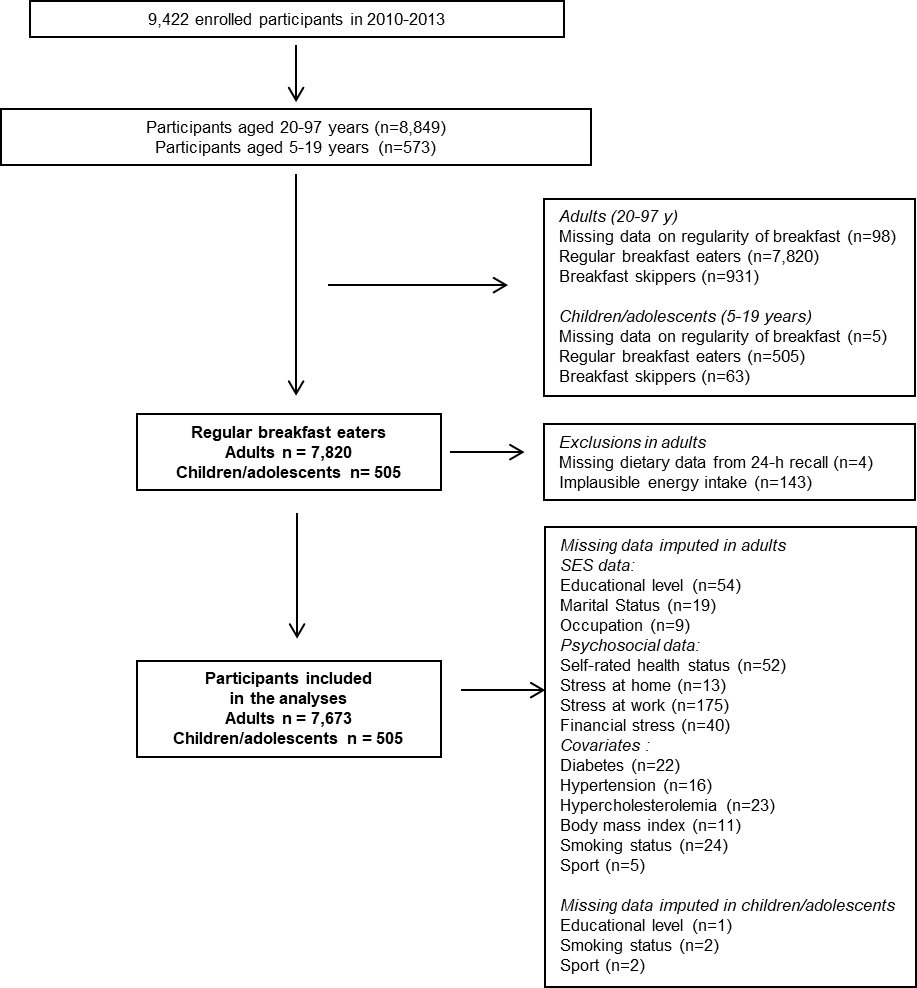
**

**Supplementary Table 1**. Criteria for scoring the Breakfast Quality Index in adult participants from the INHES Study, Italy 2010-2013

| *Items included* | Yes | No |
| --- | --- | --- |
| Cereals and derivatives consumption | +1 | 0 |
| Fruit or Vegetables consumption | +1 | 0 |
| Dairy products consumption | +1 | 0 |
| Cereal, Fruit or Vegetables and Dairy products consumption in the same meal | +1 | 0 |
| Compliance with energy intake recommendations (15-25% of total daily energy) | +1 | 0 |
| Free sugar content (<10% total daily energy divided by the number of daily eating occasion of the individual) | +1 | 0 |
| Calcium content (≥20% of daily value) | +1 | 0 |
| Saturated fat content (<10% total daily energy divided by the number of daily eating occasion of the individual) | +1 | 0 |
| Total fibre content (>25 g divided by the number of daily eating occasion of the individual) | +1 | 0 |
| Sodium content (<2000 mg divided by the number of daily eating occasions of the individual) | +1 | 0 |

**Supplementary Table 2**. Criteria for scoring the Breakfast Quality Index in children and adolescents from the INHES Study, Italy 2010-2013

| *Items included* | Yes | No |
| --- | --- | --- |
| Cereals and derivatives (bread, breakfast cereals, biscuits, bakery products) | +1 | 0 |
| Fruit and vegetables (fruit, fruit juice, vegetables) | +1 | 0 |
| Dairy products (whole and semi-skimmed milk, milk shake, yogurt, cheese) | +1 | 0 |
| Foods rich in simple sugars (sugar, jam, honey), 5% of total daily energy | +1 | 0 |
| MUFA-rich fats (olive oil, vegetable oil) | +1 | 0 |
| MUFA: SFA. Below median | +1 | 0 |
| Compliance with energy intake recommendations (20–25% of total daily energy) | +1 | 0 |
| Cereals + fruit + dairy product in the same meal | +1 | 0 |
| Ca (200-300 mg) | +1 | 0 |
| Absence of SFA and trans-rich fats (butter, margarine) | +1 | 0 |

**Supplementary Table 3**. Food groups and corresponding food items for scoring the breakfast quality indices in adults and children/adolescents from the INHES Study, Italy 2010-2013

|  |  |
| --- | --- |
|  | **Food items** |
| **Beverages** |  |
| Tea & coffee | All types of coffee (e.g. Italian espresso, mocha coffee or decaffeinated coffee) and tea (e.g. black, green) |
| Fruit juice | All types of fruit juice |
| Soft drinks | All types of water-based beverages mostly sodas (i.e. orange soda, lemon soda, cola soda, energy soda drinks, etc.) |
| Cocoa/energy drinks | Cocoa and energy drink |
| **Dairy** |  |
| Milk | All types of milk (e.g. semi-skimmed milk, whole milk, etc.) |
| Yoghurt | All types of yogurt |
| Cheese | All types of cheese |
| Other milk products | Ice cream, milk with added flavouring |
| **Meat, fish, eggs and their products** |  |
| Meat and products | All types of meat and meat products |
| Fish and products | All types of fish and fish products |
| Eggs | All types of egg and egg products |
| **Fruit and vegetables** |  |
| Fruits | All types of citrus and seasonal fruit |
| Vegetables | All types of vegetables |
| Nuts | All types of nut (e.g. pistachio, chestnuts, almonds, walnut, etc.) |
| **Cereals** |  |
| Bread and substitutes | All types of bread, packaged bread (e.g. ‘pancarrè’), bread substitutes (e.g. crackers, rusks, breadstick) |
| Breakfast cereals | All types of breakfast cereals (e.g. rice- or corn- based cereals, all bran cereals, cereals with honey, chocolate or nuts, muesli, oat cereals, millet cereals, etc.) |
| Other cereals | Rice, quinoa, millet, spelt, barley, pasta, pizza, flour. |
| **Cakes and sweets** |  |
| Cakes/pies/biscuits | Cookies, cake and pastries, croissant, chocolate |
| Snacks | All types of chocolate confectionery |
| Added sugar | All types of sweeting ingredient (i.e. white sugar, brown sugar, honeys, fructose, etc.) |
| **Added fat/oil** |  |
| Vegetable fat | Olive oil, coconut oil, corn oil, sunflower oil, soybean oil, sesame oil, peanut oil |
| Butter/Margarine | Butter and margarine |
| **Miscellaneous** |  |
| Vegetarian/vegan foods | ‘Health’ and ‘slimming’ products such as powdered or ‘fortified’ meal and dish substitutes, new plant-based meat alternatives (e.g. veggie burgers) |
| Jam/honey | Jam and honey |
| Nut spread | Nut spread |

**Supplementary Table 4.** Foods and beverages consumption at breakfast across BQI categories, in adult participants (20-97 years) from the INHES Study, Italy 2010-2013

|  |  |  | **Categories of BQI** | | |  |
| --- | --- | --- | --- | --- | --- | --- |
|  | % of consumers | Total  population | Low (0-3 points) | Medium  (4-6 points) | High (≥7 points) | p-value |
| N of participants (%) |  | 7,673 (100) | 1061 (13.8) | 6190 (80.7) | 422 (5.5) |  |
| BQI (mean ±SD) |  | 4.65 ± 1.13 | 2.91 ± 0.28 | 4.76 ± 0.72 | 7.34 ± 0.67 | <0.0001 |
| Breakfast energy intake (kcal/d) |  | 216 ±138 | 140 ±128 | 219 ±130 | 351 ±155 | <0.0001 |
| Total energy intake (kcal/d) |  | 1888 ±574 | 1687 ±534 | 1919 ±575 | 1937 ±554 | <0.0001 |
| **Beverages (g/d)** |  |  |  |  |  |  |
| Tea | 16.5 | 18.9 ± 55.9 | 33.0 ± 72.5 | 17.2 ± 52.8 | 9.7 ± 45.9 | <0.0001 |
| Coffee | 72.0 | 33.3 ± 28.2 | 35.2 ± 28.8 | 32.9 ± 27.9 | 34.7 ± 29.7 | 0.0031 |
| Fruit juice | 3.8 | 6.7 ± 36.1 | 12.6 ± 52.3 | 5.5 ± 31.7 | 9.9 ± 44.4 | <0.0001 |
| Soft drinks | 0.4 | 0.6 ± 10.7 | 1.1 ± 15.2 | 0.6 ± 10.2 | 0.0 ± 0.0 | 0.25 |
| Cocoa/energy drinks | 1.1 | 0.1 ± 3.3 | 0.0 ± 0.03 | 0.2 ± 3.7 | 0.1 ± 1.6 | 0.44 |
| **Dairy (g/d)** |  |  |  |  |  |  |
| Milk | 61.7 | 78.8 ± 72.6 | 37.1 ± 58.0 | 82.6 ± 71.3 | 127.9 ± 76.9 | <0.0001 |
| Yoghurt | 8.1 | 10.2 ± 35.1 | 2.6 ± 18.1 | 10.4 ± 35.2 | 27.1 ± 54.7 | <0.0001 |
| Cheese | 0.6 | 0.3 ± 4.6 | 0.02 ± 0.8 | 0.3 ± 4.5 | 1.4 ± 9.0 | <0.0001 |
| Other milk products | 0.5 | 0.8 ± 11.6 | 0.1 ± 2.3 | 0.7 ± 10.6 | 4.7 ± 27.9 | <0.0001 |
| **Meat, fish, eggs and their products (g/d)** |  |  |  |  |  |  |
| Meat and products | 1.1 | 0.4 ± 4.0 | 0.6 ± 4.8 | 0.3 ± 3.7 | 0.3 ± 4.9 | 0.057 |
| Fish and products | 0.1 | 0.02 ± 0.9 | 0.0 ± 0.0 | 0.02 ± 1.0 | 0.0 ± 0.0 | 0.66 |
| Eggs | 3.6 | 0.6 ± 5.1 | 0.6 ± 4.3 | 0.6 ± 4.6 | 2.0 ± 10.6 | <0.0001 |
| **Fruit and vegetables (g/d)** |  |  |  |  |  |  |
| Fruits | 6.7 | 8.6 ± 37.8 | 1.7 ± 15.8 | 7.0 ± 35.2 | 50.2 ± 73.0 | <0.0001 |
| Vegetables | 0.3 | 0.3 ± 8.0 | 0.03 ± 1.1 | 0.3 ± 7.7 | 1.7 ± 16.8 | 0.0021 |
| Nuts | 0.8 | 0.1 ± 1.4 | 0.03 ± 0.6 | 0.1 ± 1.3 | 0.5 ± 3.1 | <0.0001 |
| **Cereals (g/d)** |  |  |  |  |  |  |
| Bread and substitutes | 44.1 | 17.3 ± 31.7 | 9.6 ± 30.8 | 18.1 ± 30.7 | 24.7 ± 42.7 | <0.0001 |
| Breakfast cereals | 4.9 | 1.0 ± 5.4 | 0.3 ± 2.7 | 1.1 ± 5.6 | 1.7 ± 7.2 | <0.0001 |
| Other cereals | 0.5 | 1.5 ± 7.5 | 0.6 ± 4.8 | 1.0 ± 6.4 | 4.5 ± 19.1 | <0.0001 |
| **Cakes and sweets (g/d)** |  |  |  |  |  |  |
| Cakes/pies/biscuits | 38.7 | 17.4 ± 30.5 | 9.6 ± 25.9 | 18.4 ± 30.9 | 21.9 ± 32.6 | <0.0001 |
| Snacks | 0.3 | 0.1 ± 2.4 | 0.0 ± 0.0 | 0.1 ± 2.7 | 0.0 ± 0.0 | 0.23 |
| Added sugar | 56.7 | 6.1 ± 7.1 | 10.7 ± 7.0 | 5.5 ± 6.9 | 4.5 ± 6.5 | <0.0001 |
| **Added fat/oil (g/d)** |  |  |  |  |  |  |
| Vegetable fat | 2.2 | 0.2 ± 1.5 | 0.03 ± 0.6 | 0.2 ± 1.5 | 2.8 ± 2.0 | 0.013 |
| Butter/Margarine | 2.8 | 0.3 ± 2.1 | 0.4 ± 2.3 | 0.3 ± 2.0 | 0.6 ± 2.8 | 0.0006 |
| **Miscellaneous (g/d)** |  |  |  |  |  |  |
| Vegetarian/vegan foods | 1.4 | 2.1 ± 17.9 | 3.0 ± 22.0 | 2.0 ± 17.5 | 1.2 ± 12.3 | 0.17 |
| Jam/honey | 8.7 | 3.3 ± 19.2 | 1.4 ± 9.9 | 2.8 ± 17.8 | 15.5 ± 40.7 | <0.0001 |
| Nut spread | 0.9 | 0.2 ± 2.3 | 0.1 ± 1.9 | 0.2 ± 2.2 | 0.4 ± 4.1 | 0.068 |

Raw means.

P-values from generalized linear model adjusted for age, sex and total energy intake (kcal/d).

**Supplementary Table 5.** Foods and beverages consumption at breakfast across BQI categories, in children/adolescent (5-19 years) from the INHES Study, Italy 2010-2013

|  |  |  | **Categories of BQI** | | |  |
| --- | --- | --- | --- | --- | --- | --- |
|  | % of consumers | Total  population | Low  (0-4 points) | Medium  (5 points) | High  (≥6 points) | p-value |
| N of participants (%) |  | 505 (100) | 166 (32.9) | 191 (37.8) | 148 (29.3) |  |
| BQI (mean ±SD) |  | 4.97 ± 1.00 | 3.84 ± 0.41 | 5.00 ± 0.00 | 6.19 ± 0.54 | <0.0001 |
| Breakfast energy intake (kcal/d) |  | 287 ± 377 | 185 ±145 | 277 ± 275 | 414 ± 581 | <0.0001 |
| Total energy intake (kcal/d) |  | 2317 ± 1129 | 2384 ± 1301 | 2241 ± 848 | 2339 ± 1227 | 0.54 |
| **Beverages (g/d)** |  |  |  |  |  |  |
| Tea | 4.4 | 6.5 ± 33.0 | 6.5 ± 35.4 | 10.0 ± 39.0 | 2.0 ± 17.3 | <0.0001 |
| Coffee | 18.8 | 7.1 ± 21.3 | 7.0 ± 18.3 | 6.0 ± 25.8 | 8.4 ± 17.9 | 0.62 |
| Fruit juice | 7.9 | 16.1 ± 55.8 | 11.1 ± 46.8 | 7.3 ± 39.0 | 32.9 ± 76.5 | <0.0001 |
| Soft drinks | 1.4 | 2.5 ± 21.8 | 3.8 ± 24.2 | 3.4 ± 27.2 | 0.0 ± 0.0 | 0.25 |
| Cocoa/energy drinks | 16.8 | 2.7 ± 13.3 | 5.4 ± 22.3 | 1.5 ± 4.1 | 1.2 ± 3.8 | 0.0052 |
| **Dairy (g/d)** |  |  |  |  |  |  |
| Milk | 75.0 | 118.6 ± 86.4 | 130.4 ± 101.0 | 98.8 ± 70.9 | 130.5 ± 82.6 | 0.0005 |
| Yoghurt | 3.6 | 5.0 ± 27.1 | 7.7 ± 33.4 | 4.6 ± 26.8 | 2.5 ± 17.7 | 0.22 |
| Cheese | 0.4 | 0.2 ± 4.2 | 0.0 ± 0.0 | 0.0 ± 0.0 | 0.9 ± 7.7 | 0.10 |
| Other milk products | 0.4 | 0.5 ± 9.4 | 1.6 ± 16.4 | 0.0 ± 0.0 | 0.0 ± 0.0 | 0.16 |
| **Meat, fish, eggs and their products (g/d)** |  |  |  |  |  |  |
| Meat and products | 0.6 | 0.3 ± 3.5 | 0.0 ± 0.0 | 0.2 ± 2.3 | 0.7 ± 5.9 | 0.21 |
| Fish and products | 0.0 | 0.0 ± 0.0 | 0.0 ± 0.0 | 0.0 ± 0.0 | 0.0 ± 0.0 | - |
| Eggs | 4.5 | 0.7 ± 6.1 | 1.2 ± 5.8 | 0.6 ± 8.3 | 0.4 ± 1.6 | 0.46 |
| **Fruit and vegetables (g/d)** |  |  |  |  |  |  |
| Fruits | 4.5 | 4.0 ± 22.7 | 3.3 ± 15.6 | 4.0 ± 26.5 | 4.9 ± 24.2 | 0.81 |
| Vegetables | 0.0 | 0.0 ± 0.0 | 0.0 ± 0.0 | 0.0 ± 0.0 | 0.0 ± 0.0 | - |
| Nuts | 1.2 | 0.1 ± 1.1 | 0.3 ± 1.8 | 0.0 ± 0.4 | 0.0 ± 0.0 | 0.029 |
| **Cereals (g/d)** |  |  |  |  |  |  |
| Bread and substitutes | 18.8 | 9.3 ± 27.3 | 1.5 ± 7.5 | 15.3 ± 32.8 | 10.2 ± 31.3 | <0.0001 |
| Breakfast cereals | 9.7 | 2.1 ± 8.1 | 1.8 ± 7.6 | 3.4 ± 10.7 | 0.6 ± 3.4 | 0.0065 |
| Other cereals | 0.8 | 1.6 ± 9.8 | 2.6 ± 10.7 | 0.7 ± 8.6 | 1.6 ± 10.1 | 0.21 |
| **Cakes and sweets (g/d)** |  |  |  |  |  |  |
| Cakes/pies/biscuits | 49.3 | 36.2 ± 136.7 | 8.4 ± 36.1 | 33.7 ± 103.7 | 70.7 ± 216.0 | <0.0001 |
| Snacks | 1.0 | 0.5 ± 4.8 | 0.0 ± 0.0 | 0.7 ± 6.0 | 0.7 ± 5.8 | 0.33 |
| Added sugar | 30.1 | 4.1 ± 7.2 | 4.9 ± 8.4 | 3.0 ± 6.1 | 4.4 ± 6.8 | 0.037 |
| **Added fat/oil (g/d)** |  |  |  |  |  |  |
| Vegetable fat | 2.8 | 0.2 ± 1.4 | 0.0 ± 0.0 | 0.2 ± 1.5 | 0.5 ± 1.8 | 0.0071 |
| Butter/Margarine | 2.2 | 0.2 ± 1.6 | 0.6 ± 2.7 | 0.0 ± 0.0 | 0.0 ± 0.0 | 0.0002 |
| **Miscellaneous (g/d)** |  |  |  |  |  |  |
| Vegetarian/vegan foods | 1.2 | 1.9 ± 19.5 | 3.2 ± 21.3 | 2.3 ± 24.7 | 0.0 ± 0.0 | 0.33 |
| Jam/honey | 3.2 | 0.8 ± 5.6 | 2.1 ± 9.4 | 0.2 ± 1.4 | 0.1 ± 1.1 | 0.0008 |
| Nut spread | 5.9 | 7.1 ± 33.3 | 0.0 ± 0.0 | 5.7 ± 22.8 | 4.7 ± 16.0 | 0.0015 |

Raw means.

P-values from generalized linear model adjusted for age, sex and total energy intake (kcal/d).

**Supplementary Table 6.** Sociodemographic factors associated with Breakfast Quality Index (BQI) categories in adult participants from the INHES Study, Italy 2010-2013, by means of adjusted odds ratios (OR) with 95%CI.

|  |  | **Categories of BQI** | | |  |  |
| --- | --- | --- | --- | --- | --- | --- |
|  | Total population | Low  (0-3 points) | Medium  (4-6 points) | High  (≥7 points) | Medium vs. Low | High vs. Low |
| N of participants (%) | 7,673 | 1,0.61 (13.8) | 6,190 (80.7) | 422 (5.5%) | OR (95%CI) ^2^ | OR (95%CI) ^2^ |
| Age groups (years) |  |  |  |  |  |  |
| 20-40 | 888 (11.6) | 146 (13.8) | 710 (11.5) | 32 (7.6) | Ref. | Ref. |
| 41-65 | 4187 (54.6) | 600 (56.5) | 3380 (54.6) | 207 (49.0) | 0.98 (0.77-1.25) | 1.49 (0.92-2.41) |
| >65 | 2598 (33.8) | 315 (29.7) | 2100 (33.9) | 183 (43.4) | 1.08 (0.78-1.50) | 2.42 (1.32-4.43) |
| Sex |  |  |  |  |  |  |
| Women | 4187 (54.6) | 620 (58.4) | 3331 (53.8) | 236 (55.9) | Ref. | Ref. |
| Men | 3486 (45.4) | 441 (41.6) | 2859 (46.2) | 186 (44.1) | 0.88 (0.76-1.03) | 0.76 (0.58-0.99) |
| Geographical area |  |  |  |  |  |  |
| Northern Italy | 3220 (42.0) | 438 (41.3) | 2583 (41.7) | 199 (47.2) | Ref. | Ref. |
| Central Italy | 1318 (17.2) | 170 (16.0) | 1064 (17.2) | 84 (19.9) | 1.07 (0.88-1.30) | 1.03 (0.75-1.42) |
| Southern Italy | 3135 (40.8) | 453 (42.7) | 2543 (41.1) | 139 (32.9) | 0.94 (0.81-1.10) | 0.72 (0.55-0.95) |
| Place of residence |  |  |  |  |  |  |
| Rural | 1042 (13.6) | 175 (16.5) | 816 (13.2) | 51 (12.1) | Ref. | Ref. |
| Urban | 6631 (86.4) | 886 (83.5) | 5374 (86.8) | 371 (87.9) | 1.23 (1.02-1.49) | 1.20 (0.85-1.70) |
| Educational level |  |  |  |  |  |  |
| Up to elementary | 1393 (18.2) | 168 (15.9) | 1152 (18.6) | 73 (17.3) | Ref. | Ref. |
| Lower secondary | 1960 (25.5) | 270 (25.5) | 1595 (25.8) | 95 (22.5) | 0.89 (0.71-1.12) | 1.10 (0.75-1.61) |
| Upper secondary | 3055 (39.8) | 448 (42.1) | 2446 (39.5) | 161 (38.2) | 0.94 (0.75-1.19) | 1.30 (0.89-1.90) |
| Postsecondary | 1265 (16.5) | 175 (16.5) | 997 (16.1) | 93 (22.0) | 1.04 (0.79-1.38) | 2.02 (1.30-3.15) |
| Occupation |  |  |  |  |  |  |
| Non-manual | 2397 (31.2) | 404 (38.1) | 1865 (30.1) | 128 (30.3) | Ref. | Ref. |
| Manual | 1289 (16.8) | 161 (15.2) | 1078 (17.4) | 50 (11.8) | 1.41 (1.13-1.75) | 1.14 (0.76-1.71) |
| Housewife | 831 (10.8) | 103 (9.7) | 694 (11.2) | 34 (8.1) | 1.56 (1.19-2.04) | 1.29 (0.79-2.12) |
| Retired | 2819 (36.8) | 350 (33.0) | 2272 (36.7) | 197 (46.7) | 1.26 (0.99-1.60) | 1.51 (1.01-2.25) |
| Student | 121 (1.6) | 22 (2.0) | 95 (1.6) | 4 (1.0) | 0.97 (0.57-1.64) | 0.90 (0.27-2.99) |
| Unemployed | 216 (2.8) | 21 (2.0) | 186 (3.0) | 9 (2.1) | 2.09 (1.30-3.37) | 1.91 (0.84-4.35) |
| Marital status |  |  |  |  |  |  |
| Married /in couple | 5774 (75.2) | 778 (73.2) | 4682 (75.6) | 314 (74.4) | Ref. | Ref. |
| Unmarried | 1105 (14.4) | 182 (17.2) | 872 (14.1) | 51 (12.1) | 0.90 (0.72-1.11) | 0.93 (0.63-1.38) |
| Separated/divorced | 236 (3.1) | 21 (2.1) | 198 (3.2) | 17 (4.0) | 1.65 (1.04-2.64) | 2.02 (1.04-3.95) |
| Widowed | 558 (7.3) | 80 (7.5) | 438 (7.1) | 40 (9.5) | 0.91 (0.69-1.20) | 1.08 (0.69-1.67) |
| Smoking habit |  |  |  |  |  |  |
| Non-smoker | 4778 (62.3) | 725 (68.3) | 3799 (61.4) | 254 (60.2) | Ref. | Ref. |
| Current | 1052 (13.8) | 145 (13.7) | 862 (13.9) | 45 (10.7) | 1.13 (0.93-1.39) | 0.95 (0.65-1.38) |
| Former | 1694 (22.0) | 172 (16.2) | 1407 (22.7) | 115 (27.2) | 1.33 (1.10-1.60) | 1.63 (1.22-2.19) |
| Occasional | 149 (1.9) | 19 (1.8) | 122 (2.0) | 8 (1.9) | 1.20 (0.73-1.98) | 1.18 (0.51-2.77) |
| Sport activity |  |  |  |  |  |  |
| No | 6216 (81.0) | 894 (84.2) | 4994 (80.7) | 328 (77.7) | Ref. | Ref. |
| Yes | 1457 (19.0) | 167 (15.8) | 1196 (19.3) | 94 (2.3) | 1.43 (1.19-1.73) | 1.68 (1.24-2.26) |
| Cardiovascular disease |  |  |  |  |  |  |
| No | 741 (96.6) | 1041 (98.1) | 5966 (96.4) | 409 (96.9) | Ref. | Ref. |
| Yes | 257 (3.4) | 20 (1.9) | 224 (3.6) | 13 (3.1) | 1.63 (1.01-2.63) | 1.26 (0.61-2.62) |
| Cancer |  |  |  |  |  |  |
| No | 7420 (96.7) | 1034 (97.5) | 5978 (96.6) | 408 (96.7) | Ref. | Ref. |
| Yes | 253 (3.3) | 27 (2.5) | 212 (3.4) | 14 (3.3) | 1.33 (0.88-2.01) | 1.11 (0.57-2.16) |
| Hypertension |  |  |  |  |  |  |
| No | 5186 (67.6) | 760 (71.5) | 4153 (67.1) | 273 (64.7) | Ref. | Ref. |
| Yes | 2487 (32.4) | 301 (28.5) | 2037 (32.9) | 149 (35.3) | 1.02 (0.87-1.21) | 1.03 (0.78-1.36) |
| Hyperlipidaemia |  |  |  |  |  |  |
| No | 6000 (78.2) | 864 (81.5) | 4807 (77.7) | 329 (78.0) | Ref. | Ref. |
| Yes | 1673 (21.8) | 197 (18.5) | 1383 (22.3) | 93 (22) | 1.09 (0.91-1.30) | 0.99 (0.73-1.32) |
| Diabetes |  |  |  |  |  |  |
| No | 7078 (92.2) | 1016 (95.8) | 5663 (91.5) | 399 (94.5) | Ref. | Ref. |
| Yes | 595 (7.8) | 45 (4.2) | 527 (8.5) | 23 (5.5) | 1.97 (1.42-2.73) | 1.18 (0.69-2.02) |
| Body mass index |  |  |  |  |  |  |
| Normal weight | 3755 (48.9) | 577 (54.4) | 2960 (47.8) | 218 (51.7) | Ref. | Ref. |
| Overweight | 2916 (38.0) | 382 (36.0) | 2377 (38.4) | 157 (37.2) | 1.11 (0.95-1.29) | 1.00 (0.77-1.29) |
| Obese | 1002 (13.1) | 102 (9.6) | 853 (13.8) | 47 (11.1) | 1.40 (1.10-1.78) | 1.11 (0.74-1.65) |

Values presented are n and proportions unless otherwise indicated. ^2^ Odds ratios (OR) and 95 confidence intervals (95% CI) from a multinomial regression model including all the variables listed in the table simultaneously, and further controlled for total energy intake (kcal/d).

**Supplementary Table 7.** Psychosocial factors associated with the Breakfast Quality Index (BQI) in adult participants from the INHES Study, Italy 2010-2013, by means of adjusted odds ratios (OR) with 95%CI.

|  |  | **Breakfast Quality Index (BQI) ^1^** | |
| --- | --- | --- | --- |
| *Psychosocial factors* | N (%) | Medium vs. Low  OR (95%CI) ^2^ | High vs. Low  OR (95%CI) ^2^ |
| **Self-rated health status** |  |  |  |
| Excellent | 1211 (15.8) | Ref. | Ref. |
| Good | 4962 (64.7) | 2.59 (2.18-3.07) | 3.43 (2.38-4.94) |
| Fair | 1405 (18.3) | 2.98 (2.32-3.83) | 4.08 (2.56-6.49) |
| Poor | 95 (1.2) | 2.55 (1.29-5.02) | 3.50 (1.11-11.06) |
| **Adverse life events** |  |  |  |
| None | 6836 (89.1) | Ref. | Ref. |
| At least one | 837 (11.9) | 1.30 (1.03-1.64) | 1.42 (0.98-2.06) |
| **Stress at home** |  |  |  |
| Never | 205 (2.8) | Ref. | Ref. |
| Sometimes | 4320 (55.4) | 0.53 (0.31-0.88) | 0.50 (0.23-1.07) |
| Most of the times | 2871 (38.2) | 0.73 (0.43-1.23) | 0.68 (0.31-1.48) |
| Often/always | 277 (3.6) | 0.68 (0.36-1.27) | 0.39 (0.14-1.11) |
| **Stress at work ^a^** |  |  |  |
| Never | 149 (1.9) | Ref. | Ref. |
| Sometimes | 1503 (19.6) | 0.42 (0.20-0.84) | 0.48 (0.18-1.29) |
| Most of the times | 2077 (27.1) | 0.56 (0.27-1.14) | 0.44 (0.16-1.20) |
| Often | 326 (4.2) | 0.54 (0.25-1.17) | 0.43 (0.13-1.37) |
| Always | 142 (1.9) | 0.60 (0.25-1.45) | 1.07 (0.31-3.65) |
| Not working | 3476 (45.3) | − | − |
| **Financial stress ^b^** |  |  |  |
| Little or none | 151 (2.0) | Ref. | Ref. |
| Moderate | 4382 (57.1) | 0.79 (0.44-1.43) | 0.63 (0.27-1.47) |
| High | 2898 (37.8) | 0.48 (0.27-0.87) | 0.40 (0.17-0.94) |
| Non-responders | 242 (3.1) | − | − |

INHES, Italian Nutrition & HEalth Survey.

Values presented are n and proportions unless otherwise indicated.

^a^ Analysis run on 4,197 after exclusion of non-worker participants.

^b^ Analysis run on 7,431 after exclusion of non-responders.

^1^ Low (0-3 points); Medium (4-6 points); High (≥7 points)
^2^ Odds ratios (OR) and 95 confidence intervals (95% CI) from a multinomial regression model including age groups, sex, total daily energy intake (kcal/d), geographical area, place of residence, educational level, occupation, marital status, smoking status, sport activity, cardiovascular disease, cancer, hypertension, hypercholesterolemia, diabetes, and body mass index.

**Supplementary Table 8.** Sociodemographic factors associated with the Breakfast Quality Index (BQI) categories in children/adolescents from the INHES Study, Italy 2010-2013, by means of adjusted odds ratios (OR) with 95%CI.

|  |  | **Categories of BQI** | | |  |  |
| --- | --- | --- | --- | --- | --- | --- |
|  | Total population | Low  (0-4 points) | Medium  (5 points) | High  (≥6 points) | Medium vs. Low | High vs. Low |
| N of participants (%) | 505 | 166 (32.9) | 191 (37.8) | 148 (29.3) | OR (95%CI) ^1^ | OR (95%CI) ^1^ |
| Age groups (years) |  |  |  |  |  |  |
| 5-12 | 135 (26.7) | 45 (27.1) | 51 (26.7) | 39 (26.3) | Ref. | Ref. |
| 13-19 | 370 (73.3) | 121 (72.9) | 140 (73.3) | 109 (73.7) | 2.40 (0.98-5.88) | 1.87 (0.70-4.94) |
| Sex |  |  |  |  |  |  |
| Girls | 239 (47.3) | 77 (46.3) | 90 (47.1) | 72 (48.6) | Ref. | Ref. |
| Boys | 266 (52.7) | 89 (53.6) | 101 (52.9) | 76 (51.4) | 0.91 (0.59-1.42) | 0.86 (0.54-1.37) |
| Geographical area |  |  |  |  |  |  |
| Northern Italy | 107 (21.2) | 24 (14.5) | 41 (21.5) | 42 (28.4) | Ref. | Ref. |
| Central Italy | 44 (8.7) | 21 (12.6) | 15 (7.8) | 8 (5.4) | 0.39 (0.16-0.93) | 0.22 (0.08-0.59) |
| Southern Italy | 354 (70.1) | 121 (72.9) | 135 (70.7) | 98 (66.2) | 0.75 (0.42-1.36) | 0.54 (0.29-0.98) |
| Place of residence |  |  |  |  |  |  |
| Rural | 61 (12.1) | 24 (14.5) | 25 (13.1) | 12 (8.1) | Ref. | Ref. |
| Urban | 444 (87.9) | 42 (85.5) | 166 (86.9) | 136 (91.9) | 1.03 (0.53-2.01) | 1.62 (0.73-3.55) |
| Educational level |  |  |  |  |  |  |
| Up to elementary | 180 (35.6) | 54 (32.5) | 73 (38.2) | 53 (35.8) | Ref. | Ref. |
| Lower/upper secondary | 325 (64.4) | 112 (67.5) | 118 (61.8) | 95 (64.2) | 0.41 (0.17-0.96) | 0.50 (0.20-1.29) |
| Smoking habit |  |  |  |  |  |  |
| Non-smoker | 448 (88.7) | 142 (85.5) | 173 (90.6) | 133 (89.9) | Ref. | Ref. |
| Current/former/occasional | 57 (11.3) | 24 (14.5) | 18 (9.4) | 15 (10.1) | 0.69 (0.35-1.37) | 0.76 (0.37-1.55) |
| Sport activity |  |  |  |  |  |  |
| No | 170 (33.7) | 62 (37.3) | 61 (31.9) | 47 (31.8) | Ref. | Ref. |
| Yes | 335 (66.3) | 104 (62.7) | 130 (68.1) | 101 (68.2) | 1.35 (0.85-2.16) | 1.17 (0.71-1.93) |
| Body mass index |  |  |  |  |  |  |
| Normal weight | 419 (83.0) | 137 (82.5) | 156 (81.7) | 126 (85.1) | Ref. | Ref. |
| Overweight/Obese | 86 (17.0) | 29 (17.5) | 35 (18.3) | 22 (14.9) | 1.18 (0.67-2.10) | 0.96 (0.51-1.81) |

INHES, Italian Nutrition & HEalth Survey. Values presented are n and proportions unless otherwise indicated. ^1^ Odds ratios (OR) and 95 confidence intervals (95% CI) from a multinomial regression model including all the variables listed in the table simultaneously, and further controlled for total energy intake (kcal/d).
